# Clinical Outcomes After Dental Surgery with Two Antiseptic Protocols

**DOI:** 10.3390/dj12120389

**Published:** 2024-11-28

**Authors:** Silvia D’Agostino

**Affiliations:** 1 Department of Medical, Oral and Biotechnological Sciences, University G. d’Annunzio, 66100 Chieti, Italy; silvia.dagostino@unich.it; 2 Complex Unit of Odontostomatology, University A. Moro, 70124 Bari, Italy

**Keywords:** chlorhexidine, cetylpyridinium chloride, vitamin B3, antiseptic, oral surgery, dental surgery

## Abstract

**Background:** Little has been written in the literature about the clinical comparison between the single use of chlorhexidine (CHX) and its combination with cetylpyridinium chloride (CPC). The purpose of this study is to compare the clinical effectiveness of two at-home antiseptic regimens. **Methods**: Healthy subjects scheduled for dental surgery were enrolled. After the surgery, patients were randomly allocated to the first group (group A), which received a manual ultrasoft toothbrush (Mentadent Professional^®^), an antiseptic toothpaste with 0.12% CHX and Vitamin B3 (Mentadent Professional Azione Intensiva Gengive^®^), and an antiseptic mouthwash with 0.12% CHX and 0.07% CPC. The second group (group B) solely received an antiseptic mouthwash with 0.2% CHX in conjunction with an anti-discoloration system (Curasept ADS/DNA^®^) and were encouraged to use their usual toothbrush and toothpaste. Patients were instructed to use the products twice a day and to rinse for 30 s. On day 7, patients were examined for the early healing score (EHS), visual plaque index (VPI) of the sutures, numerical rating scale (NRS), and mouthwash taste. **Results**: Group A showed a statistically significant level of EHS and taste satisfaction. VPI and NRS were different but not significant among the studied groups. **Conclusions**: The regimen based on CHX used in conjunction with vitamin B3 in the toothpaste and CPC in the mouthwash resulted in superior clinical outcomes and satisfaction compared to CHX alone.

## 1. Introduction

Oral surgery encompasses a wide range of procedures performed within the oral cavity. These procedures can involve tooth extraction, wisdom tooth removal, implant placement, jaw surgery, and other corrective or restorative interventions. While these procedures aim to improve oral health and function, they inevitably cause tissue disruption and initiate an inflammatory response [1]. Inflammation is a complex biological process that serves a crucial role in wound healing by isolating and eliminating pathogens, promoting tissue repair, and restoring homeostasis. However, in the context of oral surgery, the inflammatory response can also contribute to postoperative discomfort, swelling, and potential complications [2]. Understanding the mechanisms of inflammation after oral surgery is essential to optimize patient recovery and develop novel therapeutic strategies.

The surgical procedure itself triggers a cascade of inflammatory events. Tissue injury leads to the release of various inflammatory mediators, including cytokines, chemokines, and vasoactive substances. These mediators promote the recruitment and activation of immune cells, such as neutrophils, macrophages, and lymphocytes, to the surgical site. Neutrophils are the first line of defense, engulfing and destroying bacteria and debris. Macrophages play a pivotal role in phagocytosis, debris clearance, and tissue repair by releasing growth factors and stimulating angiogenesis (new blood vessel formation). Lymphocytes participate in the adaptive immune response, providing long-term immunological memory [3].

Vasoactive substances cause vasodilation (the widening of blood vessels) and increased blood flow, leading to redness and swelling at the surgical site. This increased blood flow delivers essential immune cells, oxygen, and nutrients for healing [4]. However, excessive inflammation can cause uncontrolled vasodilation and edema, contributing to facial puffiness and discomfort [5].

Macrophages orchestrate the deposition of collagen, a key structural protein, to rebuild damaged tissues. However, prolonged or dysregulated inflammation can lead to excessive scar tissue formation, which can impair function and aesthetics [6].

Postoperative inflammation after oral surgery typically manifests as swelling, pain, and tenderness at the surgical site. The degree of swelling can vary depending on the type and complexity of the surgery. Swelling usually peaks within 2–3 days and gradually subsides over the following week. Pain is another well-documented consequence of surgical intervention, and its management is typically achieved through the administration of analgesic medications [7,8,9].

While inflammation is a natural part of healing, excessive or prolonged inflammation can lead to complications. One such complication is referred to as dry socket, a painful condition that occurs when the blood clot protecting the exposed bone socket dissolves prematurely [10].

Currently, the management of postoperative inflammation primarily relies on pharmacological interventions. Nonsteroidal anti-inflammatory drugs (NSAIDs) are the cornerstone of therapy, acting by inhibiting the enzymes responsible for the production of prostaglandins, key inflammatory mediators. NSAIDs effectively alleviate pain and reduce swelling [11]. However, NSAIDs can have gastrointestinal side effects and may not be suitable for all patients. Corticosteroids are another class of medications used to suppress inflammation. They are typically used for short periods due to potential side effects like delayed wound healing and increased susceptibility to infection [12].

Other strategies to reduce post-operative inflammation could be the use of platelet-rich plasma and platelet-rich fibrin [13] and the development of natural polymers such as chitosan [14].

Maintaining oral hygiene after oral surgery procedures is crucial for a successful outcome; additionally, it prevents the administration of the antibiotic therapy [15]. Disruption of the oral tissues during surgery creates a vulnerable environment susceptible to bacterial colonization and potential infection. This can delay healing, increase discomfort, and even lead to complications [16]. The maintenance of sterile procedures during oral surgeries is paramount for optimal healing and infection prevention. A meticulously clean operating environment minimizes the risk of microbial contamination, thereby promoting a conducive environment for wound healing. Chlorhexidine (CHX) has emerged as an efficient agent for preventing infections owing to its potent antimicrobial properties. Additionally, rigorous disinfection protocols are essential to eradicate pathogens and mitigate their transmission. Various methods, including ultraviolet C (UVC) radiation, gaseous ozone, and liquid chemical disinfectants, offer effective strategies for eliminating microorganisms from surfaces and equipment. By implementing these comprehensive infection control measures, healthcare providers can significantly reduce the incidence of healthcare-associated infections and ensure optimal patient outcomes.

Oral antiseptics have emerged as a valuable tool in the postoperative care regimen. These agents offer a targeted approach to reduce the bacterial load and promote a more favorable healing environment within the oral cavity [17].

**Figure 1:**
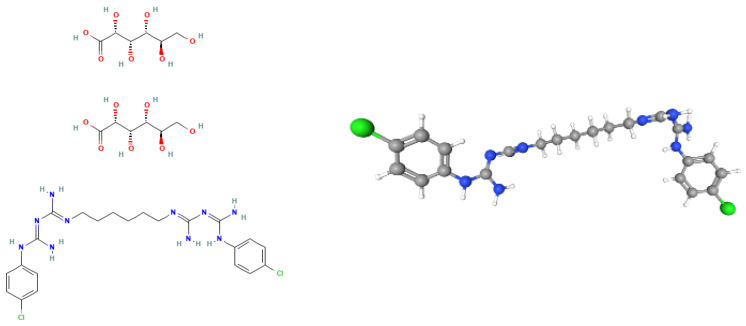
On the left side, the 2D structure of CHX. On the right side, the 3D structure of CHX.

CHX has been a mainstay in oral antisepsis for decades. Its broad-spectrum antimicrobial activity effectively targets a wide range of bacteria associated with oral infections [18]. It has the following molecular formula: C_22_H_30_C_l2_N_10_. 2C_6_H_12_O_7_ C_34_H_54_C_l2_N_10_O_14_. Its 2D and 3D structures are provided in Figure 1.

Studies have demonstrated its effectiveness in reducing plaque formation and promoting gingival healing after periodontal procedures [19]. However, CHX can have drawbacks, including potential staining of the teeth and taste alterations [20]. Cetylpyridinium chloride presents itself as an alternative antiseptic option. It possesses antimicrobial properties and demonstrates efficacy in reducing plaque and preventing gingivitis [21]. Additionally, CPC may be less likely to cause taste disturbances compared to CHX [22]. Despite the established benefits of both antiseptics, the optimal use of CHX and CPC in the context of oral surgery remains a topic of ongoing research.

This study aims to evaluate the clinical outcomes of two different antiseptic protocols after oral surgical procedures of tooth extraction with a flap elevation, single implant placement, and odontoma removal.

## 2. Materials and Methods

This study was carried out in accordance with the principles of the Declaration of Helsinki, approved by the Local Institutional Review Board active at the University G. d’Annunzio of Chieti, Italy (Protocol 136/02-04-2024). All the patients included released a detailed written informed consent.

### 2.1. Patients Selection

Patients from the dental clinic of the University G. d’Annunzio of Chieti, Italy, without age and sex limitations, were included in the present monocenter randomized study.

Inclusion criteria were as follows:

- Scheduled dental surgery, tooth extraction with a flap elevation, single implant placement, and odontoma removal;
- Absence of periodontitis;
- Absence of allergy to chlorhexidine and/or cetylpyridinium chloride;
- Good general health, in particular no disease that could potentially delay wound healing;
- No intake of any medical drug that could influence the outcome of the study, such as immunosuppressors (azathioprine, everolimus, mycophenolic acid).

Pregnant women, patients with periodontitis, and diabetics were excluded.

### 2.2. Subject Allocation

Subjects were randomly allocated to one of the treatment sequences by a computer software (Urbaniak, G. C., & Plous, S. (2013). Research Randomizer, Version 4.0) [23]. Allocation to one of two treatment sequences was exclusively discovered by the dentist just before the delivery of the antiseptic treatment regimen.

Patients were randomly allocated to the first group (group A), which received a manual ultrasoft toothbrush (Mentadent Professional^®^, Unilever Italia, Rome, Italy) with 0.08 mm diameter bristles, an antiseptic toothpaste with 0.12% chlorhexidine and Vitamin B3 (Mentadent Professional Azione Intensiva Gengive^®^), and an antiseptic mouthwash with 0.12% chlorhexidine and 0.07% cetylpyridinium chloride.

The second group (group B) solely received an antiseptic mouthwash with 0.2% CHX in conjunction with an anti-discoloration system (ADS, Curasept ADS/DNA^®^, Curasept, Varese, Italy) and were encouraged to use their usual toothbrush and toothpaste.

Patients were instructed to use the products twice a day and to rinse for 30 s.

The products for the two different regimens were provided at no cost to the patients.

### 2.3. Treatment Protocol

Two weeks prior to the surgery, all subjects were subject to professional dental hygiene treatments followed by proper instructions and motivation to achieve healthy gingival tissues prior to the study beginning. At baseline, after the surgical procedure, all subjects received the products for the regimen to perform at home.

All patients received post-operative prescriptions for betamethasone with the following posology: 4 mg from day 1 to 3, 2 mg from day 4 to 7. Antibiotics were not prescribed in accordance with the 2021 American Heart Association (AHA) Guidelines [24] for subjects with good general health.

### 2.4. Clinical Measurements

On day 7 after the dental surgery, patients were examined for the early healing score (EHS), as stated by Marini L. et al. [25], and for the visual plaque index, as per Ainamo J. and Bay I. [26], through observation of the sutures. Finally, a questionnaire for the pain assessment using the numerical rating scale (NRS) by Downie WW et al. [27] and for taste opinion was distributed. The patient was asked to indicate a number from 0, for a strong unpleasant taste, to 5, for extremely pleasant taste.

### 2.5. Statistical Analysis

The data were assessed for normality using the Shapiro–Wilk test. If the data were normal, they were analyzed using the parametric Student’s *t* test. If the data were not normal, they were analyzed using the non-parametric Kruskal–Wallis test. The level of significance was set to *p*-values lower than 0.05.

## 3. Results

**Figure 2:**
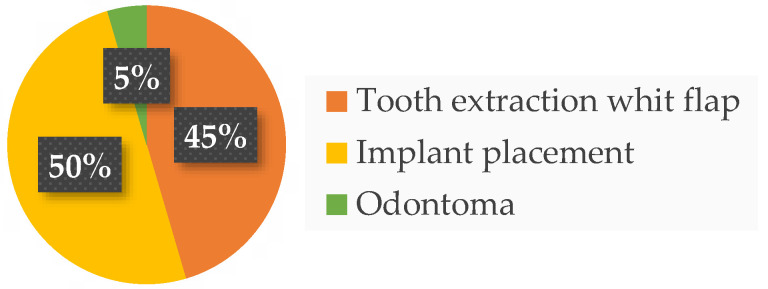
Distribution of surgical procedures.

**Figure 3:**
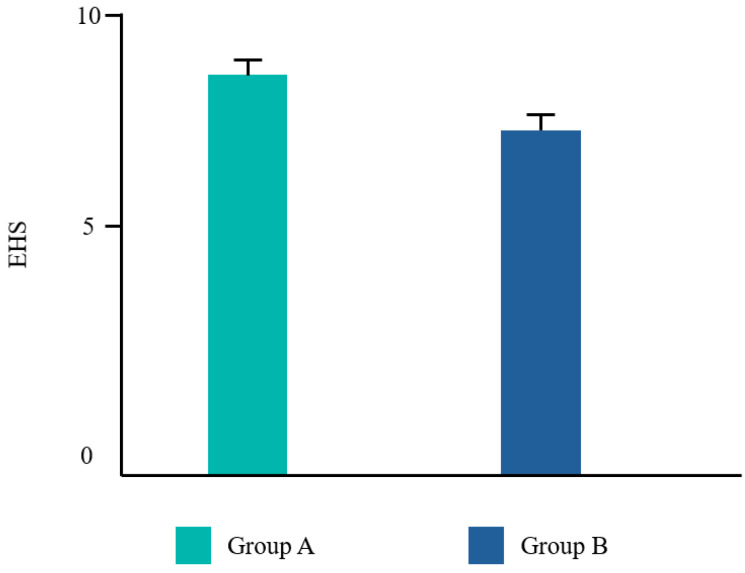
Mean values for the early healing score (EHS).

**Figure 4:**
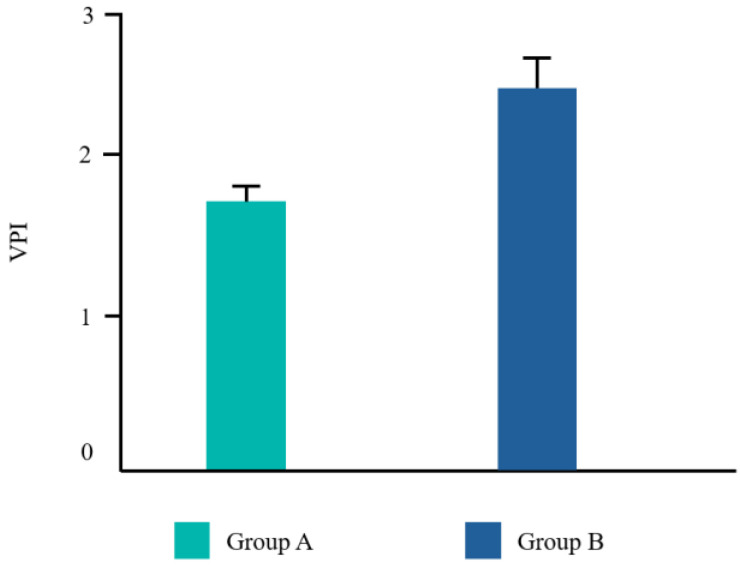
Mean values for the visual plaque index (VPI).

**Figure 5:**
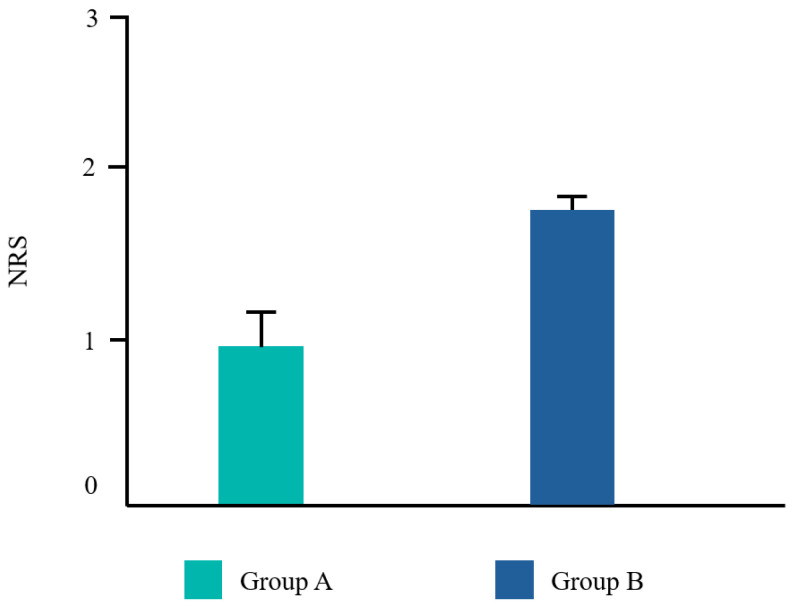
Mean values for the numerical rating scale (NRS).

**Figure 6:**
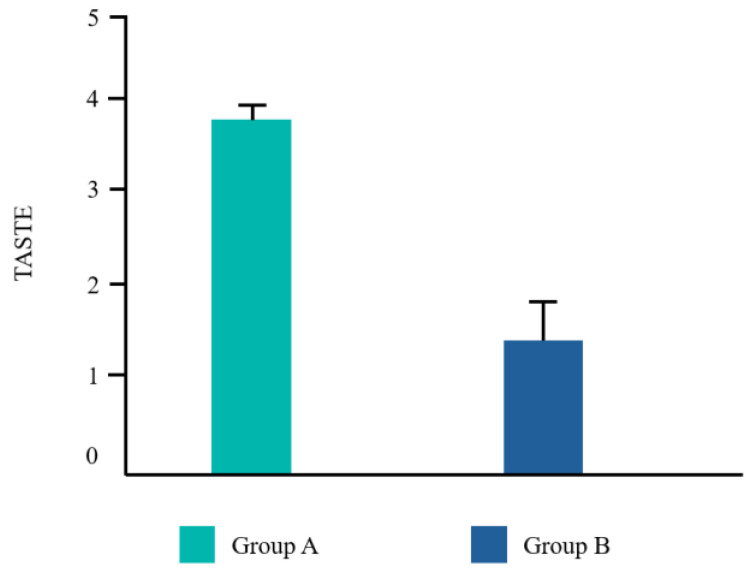
Mean values for taste.

Twenty-two patients from the dental clinic of the University G. d’Annunzio of Chieti, Italy, 15–68 years old (mean age 39.2 ± 14.19 years) were included, 45% (10/22) were male and 55% (12/22) were female. All patients completed the study. No adverse events related to the treatments used were reported by any of the participants. Ten patients underwent a tooth extraction with flap elevation, 11 underwent a single implant placement, and one underwent the removal of an odontoma (Figure 2). Frequency distributions of all the analyzed parameters showed a normal profile of the data. Each variable was reported as follows (Figure 3, Figure 4, Figure 5 and Figure 6).

The main findings are summarized in Table 1.

The Student’s *t* test was significant for EHS and taste. No difference was found for VPI and NRS (Table 2).

### Limitation of the Study

The protocol shown presents some limitations. The first issue was that group B did not receive an antiseptic toothpaste like group A. The second issue was that the EHS has some intrinsic subjective aspects like every clinical index for wound healing. An objective parameter might have been the histological analysis of a tissue sample from the wound site. Obviously, this invasive procedure would not have been endorsed by the ethics committee without a clinical/therapeutical reason. Finally, the statistics did not involve confounding and bias analysis. For all these reasons, the data should be interpreted with caution.

## 4. Discussion

The present study aims to provide insights into different antiseptic molecules by evaluating the clinical efficacy of two at-home antibacterial regimens following dental surgery, due to the fact that CHX is a well-established antiseptic molecule in oral healthcare, while limited research exists comparing its single use to combinations with CPC.

CHX is a broad-spectrum antiseptic agent in dentistry, demonstrably effective in reducing plaque and gingivitis [19]. However, its clinical utility is not straightforward in the context of both periodontitis [28] and peri-implant mucositis [29]. While generally well-tolerated, CHX can induce a range of adverse effects that warrant consideration, particularly in relation to long-term use. One of the most commonly reported drawbacks of CHX is taste alteration. Studies have documented a metallic or bitter taste sensation following CHX use, potentially impacting patient compliance [30,31]. This can be particularly concerning for patients already experiencing taste disturbances such as dysgeusia. Furthermore, CHX can trigger hypersensitivity reactions, manifesting as localized burning or irritation of the oral mucosa [30]. While these reactions are typically mild and transient, they can deter patients from continued use. Additionally, prolonged CHX exposure has been linked to the staining of the teeth and tongue, especially when used in a 0.2% concentration, presenting an aesthetic concern for some patients [29]. To overcome these problems, recent studies have suggested the use of platelet-rich plasma and platelet-rich fibrin [13], as well as the use of natural polymers such as chitosan [14], which may have a potentially synergistic effect when used in combination with CPC. This synergy translates to enhanced antimicrobial activity compared to the individual agents alone [31,32]. The mechanism underlying this synergy remains under investigation, but several hypotheses have been proposed. One possibility involves the disruption of microbial cell membranes. Both CHX and CPC possess cationic properties that electrostatically attract the negatively charged bacterial membranes. CHX is theorized to primarily target the cytoplasmic membrane, leading to cell death [33]. CPC, on the other hand, might disrupt the outer membrane, facilitating CHX penetration and amplifying its effect [34,35].

In the present study, subjects in group A exhibited statistically significant improvements in both the EHS and taste satisfaction. This observation suggests that the comprehensive regimen employed by group A, which incorporated CHX alongside vitamin B3 in the toothpaste and cetylpyridinium chloride CPC in the mouthwash, yielded superior clinical outcomes and patient satisfaction compared to the regimen used by group B, which solely relied on a higher concentration of CHX in the mouthwash. Vitamin B3, also known as niacinamide, is a water-soluble essential nutrient that plays a crucial role by strengthening the cellular barrier, thus enhancing the ceramide biosynthesis [36]. According to Wessels Q. et al. [37], fibroblast collagen synthesis increased, alongside cellular migration and proliferation, after a topical application of niacinamide. Adding this molecule to a fluoride toothpaste may be useful to increase the permeability barrier of the oral mucosa [38].

Regarding the mouthwash employed, the combination of CHX and CPC allows to use lower concentrations of CHX, reducing the risk of side effects. This leads to a more pleasant flavor which may enhance patient compliance with the prescription [37].

Even though VPI was lower in group A compared to group B, the results were not statistically significant. This may be explained by the fact that group B still used an antiseptic mouthwash which may prevent the dental biofilm from spreading. The same can be assumed for NRS. Our results are in line with those by Guerra F. et al. [39], for the authors reported that CHX with ADS showed a limited ability to reduce bacterial plaque and gingival bleeding. Additionally, the authors concluded that the anti-stain molecule, added to the formulation, did not reduce pigmentation compared to mouthwashes without it after a spectrophotometric assessment. Finally, Li et al. [19] found that CHX with ADS did not completely eliminate the side-effect of staining.

The present study reports some limitations. The first limit is represented by the fact that group B did not receive an antiseptic toothpaste but was encouraged to use their habitual toothbrush and dentifrice. The second limit is the small sample size. Finally, the results were not analyzed according to the different surgical procedures received by the participants. It is important to note that variations in the type of intervention can significantly impact post-surgical outcomes.

Despite the limitations of the present study, CHX remains the cornerstone of the antiseptic management of post-surgical inflammation in dentistry. This study suggests a potential synergistic interaction between CHX and CPC that could offer a promising avenue for optimizing post-surgical care. The observed improvements in patient satisfaction in relation to the CHX–CPC combination highlight the importance of addressing potential adverse effects associated with the traditional CHX regimens. Future research with larger sample sizes and longer follow-up periods is necessary to definitively establish the efficacy and safety profile of CHX–CPC combinations in post-surgical dental management.

## 5. Conclusions

The implementation of a well-designed antiseptic regimen plays a pivotal role in the post-surgical management of inflammatory processes following dental procedures. This regimen likely exerts its effects through a multifaceted approach, including the reduction of the burden of pathogenic bacteria that can contribute to post-surgical complications. Both tested groups showed good plaque reduction. Group A was associated with a better EHS, maybe owing to the more pleasant taste of the mouthwash used. The employment of a protocol based on a manual ultrasoft toothbrush, an antiseptic toothpaste with 0.12% chlorhexidine with vitamin B3, and an antiseptic mouthwash with 0.12% chlorhexidine and 0.07% cetylpyridinium chloride proved to be effective in the management of the flogosis that follows oral surgery.

## Figures and Tables

**Table 1:** Results for the early healing score (EHS), visual plaque index (VPI), and numerical rating scale (NRS). Values are presented as the mean ± standard deviation.

	EHS	VPI	NRS	Taste
**Group A**	8.5 ± 1.29	1.7 ± 1.27	0.9 ± 1.32	3.7 ± 1.04
**Group B**	6.8 ± 0.75	2.5 ± 0.68	1.7 ± 1.34	1.5 ± 0.07

**Table 2:** Student’s *t* test results in relation to early healing score (EHS), visual plaque index (VPI), and numerical rating scale (NRS).

	EHS	VPI	NRS	Taste
***p*-value**	0.002	0.115	0.128	0.000
**Significance**	Yes	No	No	Yes

## Data Availability

The data supporting the reported results can be obtained from the University G. d’Annunzio, 31, Via Dei Vestini, 66100 Chieti, Italy.
